# Nuclear receptor NR4A1 is a tumor suppressor down-regulated in triple-negative breast cancer

**DOI:** 10.18632/oncotarget.17532

**Published:** 2017-04-29

**Authors:** Hongmei Wu, Jiong Bi, Yan Peng, Lei Huo, Xiaobin Yu, Zhihui Yang, Yunyun Zhou, Li Qin, Yixiang Xu, Lan Liao, Yang Xie, Orla M. Conneely, Jos Jonkers, Jianming Xu

**Affiliations:** ^1^ Department of Molecular and Cellular Biology, Baylor College of Medicine, Houston, TX 77030, USA; ^2^ Department of Pathology, UT Southwestern Medical Center, Dallas, TX 75390, USA; ^3^ Department of Pathology, UT MD Anderson Cancer Center, Houston, TX 77030, USA; ^4^ Institute for Cancer Medicine, School of Basic Medical Sciences, and Department of Pathology, Xinan Medical University, Luzhou, Sichuan 646000, China; ^5^ Simmons Comprehensive Cancer Center, UT Southwestern Medical Center, Dallas, TX 75390, USA; ^6^ Netherlands Cancer Institute, Antoni van Leeuwenhoek Hospital, 1066 CX Amsterdam, Netherlands; ^7^ Current address: College of Life Sciences, Shaanxi Normal University, Xi’an, Shaanxi 710062, China; ^8^ Current address: Departments of General Surgical Laboratory, The First Affiliated Hospital of Sun Yat-Sen University, Guangzhou 510080, China; ^9^ Current address: Department of Data Science, University of Mississippi Medical Center, Jackson, MS 39216, USA

**Keywords:** triple-negative breast cancer, gene expression, nuclear receptor, NR4A1, tumor suppressor

## Abstract

The nuclear receptor (NR) superfamily contains hormone-inducible transcription factors that regulate many physiological and pathological processes through regulating gene expression. NR4A1 is an NR family member that still does not have an identified endogenous ligand, and its role in cancer is also currently unclear and controversial. In this study, we aimed to define the expression profiles and specific role of NR4A1 in the highly malignant triple-negative breast cancer (TNBC), which still lacks available targeted therapies. Bioinformatic analysis revealed a decrease of NR4A1 mRNA expression in human TNBC samples. Semi-quantitative analysis of NR4A1 protein expression by immunohistochemistry also identified a progressive NR4A1 reduction during the development of mouse basal-like mammary tumors and a significant NR4A1 downregulation in human TNBC samples. Furthermore, the expression levels of NR4A1 in human TNBC were negatively associated with tumor stage, lymph node metastasis and disease recurrence. Moreover, ectopic expression of NR4A1 in MDA-MB-231, a TNBC cell line with little endogenous NR4A1, inhibited the proliferation, viability, migration and invasion of these cells, and these inhibitions were associated with an attenuated JNK1–AP-1–cyclin D1 pathway. NR4A1 expression also largely suppressed the growth and metastasis of these cell-derived tumors in mice. These results demonstrate that NR4A1 is downregulated in TNBC and restoration of NR4A1 expression inhibits TNBC growth and metastasis, suggesting that NR4A1 is a tumor suppressor in TNBC.

## INTRODUCTION

Breast cancer is the most common cancer type in women and poses a major threat to woman's health. Triple-negative breast cancer (TNBC), which do not express estrogen receptor α (ERα), progesterone receptor (PR) and human epidermal growth factor receptor 2 (HER2), accounts for 15% of all breast carcinomas and about 70–80% of basal-like breast cancer [1, 2]. Because TNBC does not express ERα and HER2, patients with TNBC cannot benefit from the currently available endocrine and anti-HER2 therapies. Instead, the mainstay of systemic treatment for TNBC patients is chemotherapy. However, the response of many TNBC patients to chemotherapy is poor [3–5]. TNBC that fail to response to initial therapy has a high risk of recurrence and exhibits poor prognosis [1, 4]. Therefore, there is an urgent need to develop targeted therapies for treating TNBC. Identification and characterization of key molecules that control the growth, progression and metastasis of TNBC cells should facilitate the selection of drug targets and the development of new therapeutic strategies.

Nuclear hormone receptors (NRs) such as ERα and androgen receptor (AR) are hormone-inducible transcription factors. These NRs regulate numerous physiological and pathological processes by regulating their target gene expression through hormone-induced DNA binding and coregulator recruitments [6, 7]. Since most hormones or ligands of NRs are small, cell membrane-permeable hydrophobic molecules, many synthetic agonists and antagonists of NRs have been developed and widely used to treat various diseases. For example, tamoxifen, a selective estrogen receptor modulator, and enzalutamide, an androgen receptor inhibitor, are respectively used to treat breast and prostate cancers, while dexamethasone, an agonist of glucocorticoid receptor, is widely used to control inflammation. To date, multiple NRs including nuclear receptor 4A1 (NR4A1, also known as Nur77, TR3 or NGFIB) still do not have an identified endogenous hormone or ligand; therefore, these NRs have not been used as therapeutic targets. It is possible that some of these NRs may play an important role in TNBC, providing a hope for finding a new druggable target for inhibiting TNBC growth and metastasis.

Many kinds of extracellular stimuli such as growth factors, prostaglandins, calcium, cytokines and neurotransmitters can induce NR4A1 expression in cells [8]. NR4A1 can bind to a NR4A response element (AAAGGTCA) as a monomer or a Nur response element (TGATATTTX6AAAGTCCA) as homodimer or heterodimer with retinoid X receptor (RXR) to regulate gene transcription [8]. Together with other NR4A subfamily members, NR4A1 regulates many physiological functions involving the central nervous system, steroidogenesis, inflammation, vascular smooth muscle and metabolism [8]. Interestingly, the overall reported role of NR4A1 in cancer is paradoxical in the literature. On one hand, several studies have reported NR4A1 as a tumor suppressor. For example, double knockout of Nr4a1 and Nor1 genes in mice causes acute myeloid leukemia (AML) [9]. Some lymphomas have a decrease in NR4A1 expression, and overexpression of NR4A1 in certain lymphoma cells induces apoptosis and inhibits the growth of these cell-derived tumors in mice [10]. NR4A1 also slows down the growth of LNCaP prostate cancer cells [11]. Ectopic expression of NR4A1 in ZR-75-1 breast cancer cells with ERα expression and PMC42 breast cancer cells with progenitor characteristics can inhibit cell migration although proliferation and apoptosis of these cells are unaffected [12]. On the other hand, several studies have reported NR4A1 as an oncogenic driver. For example, NR4A1 expression is elevated in pancreatic, bladder, lung and colon cancers [13–16]. Knockdown of NR4A1 in lung and pancreatic cancer cells decreases their growth and increases their apoptosis [15, 16]. One study reported that about 50% of breast tumors showed NR4A1 overexpression; TNF-α-induced NR4A1 expression in MCF-7 ERα-positive breast cancer cells plays an anti-apoptotic role; and ectopic expression of NR4A1 also promotes MCF-7 cell growth [17]. Another study observed NR4A1 overexpression in breast tumors with high immune infiltration and poor prognosis and showed a promoting role of NR4A1 in the invasion and metastasis of breast cancer cells by activating TGFβ signaling [18]. These controversial reports regarding NR4A1 in cancer highlights the possibility that the complex role of NR4A1 in cancer may be specific to the types or subtypes of cancers.

Since the role of NR4A1 in TNBC has not been specifically investigated, this study aims to define the expression profiles and specific role of NR4A1 in this highly malignant cancer. We report that NR4A1 protein expression is decreased in the mouse basal-like mammary tumors during the tumor progression process and in a large proportion of human TNBC tumors. The low expression of NR4A1 protein in human TNBC samples is associated with advanced tumor stage, lymph node metastasis and disease recurrence. We also demonstrates that expression of NR4A1 in MDA-MB-231 cells significantly inhibits the proliferation, viability, migration and invasion of these cells in culture and the growth and metastasis of these cell-derived tumors in mice. Our findings suggest that NR4A1 is a tumor suppressor in TNBC.

## RESULTS

### The expression of NR4A1 mRNA is downregulated in TNBC

**Figure 1:**
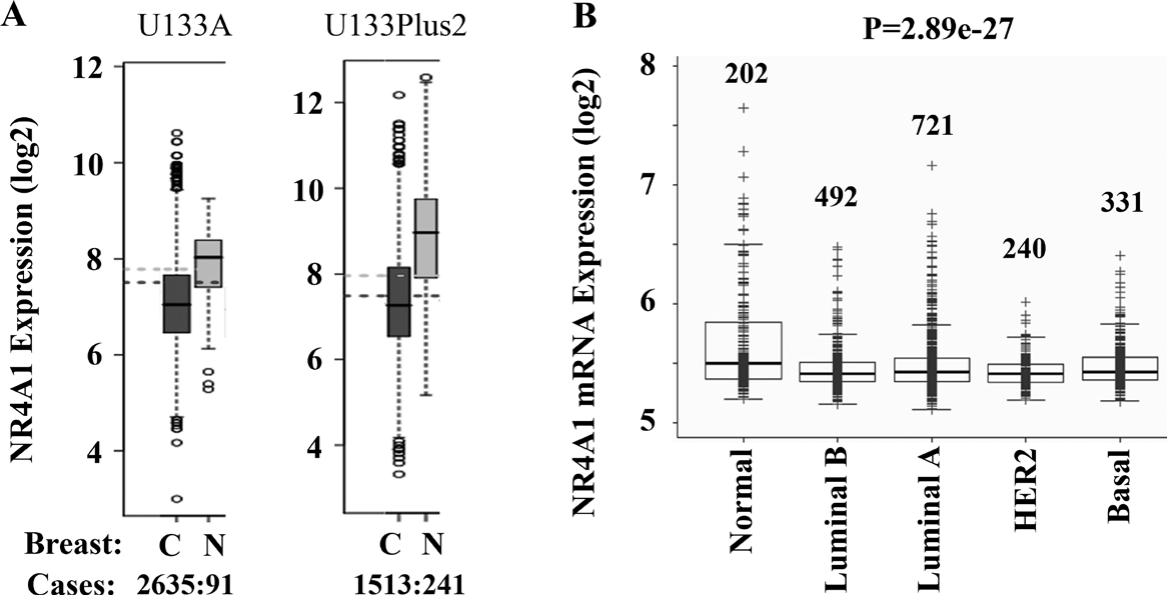
Bioinformatic analysis of NR4A1 expression in human breast cancer and normal breast tissue (**A**) Analyses of U133A and U133Plus2 datasets in the GENT (Gene Expression Across Normal and Tumor Tissue) Database revealed a significant decrease in NR4A1 mRNA expression in breast cancer tissues versus normal tissues. The case number in each group is indicated. C cancer; N, normal. (**B**) Analysis of the Metabric dataset with 1986 samples showed a significant decrease in NR4A1 mRNA expression in human breast cancers versus normal breast tissue. The number of samples in each group and the *p* value are indicated.

As our first step to explore the role of NR4A1 in cancer, we compared the expression levels of NR4A1 mRNA in human breast cancer and normal breast tissues by analyzing the data sets in the database of Gene Expression Across Normal and Tumor Tissue (GENT) [19]. Analysis of both U133A expression dataset from 2635 breast cancer and 91 normal breast tissue specimens and U133Plus2 expression dataset from 1513 breast cancer and 241 breast normal tissue specimens revealed that NR4A1 expression was significantly downregulated in breast cancers versus normal breast tissues (Figure 1A). Analysis of the Metabric dataset obtained from 1986 specimens [20] also identified a significant decrease in NR4A1 mRNA expression in luminal A/B, HER2-positive and basal-like breast cancers when compared with normal breast tissues (Figure 1B). Most of the basal-like breast cancers in the study are TNBC. These analyses suggest that the NR4A1 gene transcript is downregulated in human breast cancer including TNBC.

### NR4A1 protein is progressively downregulated during the progression of the basal-like mouse mammary tumors

**Figure 2:**
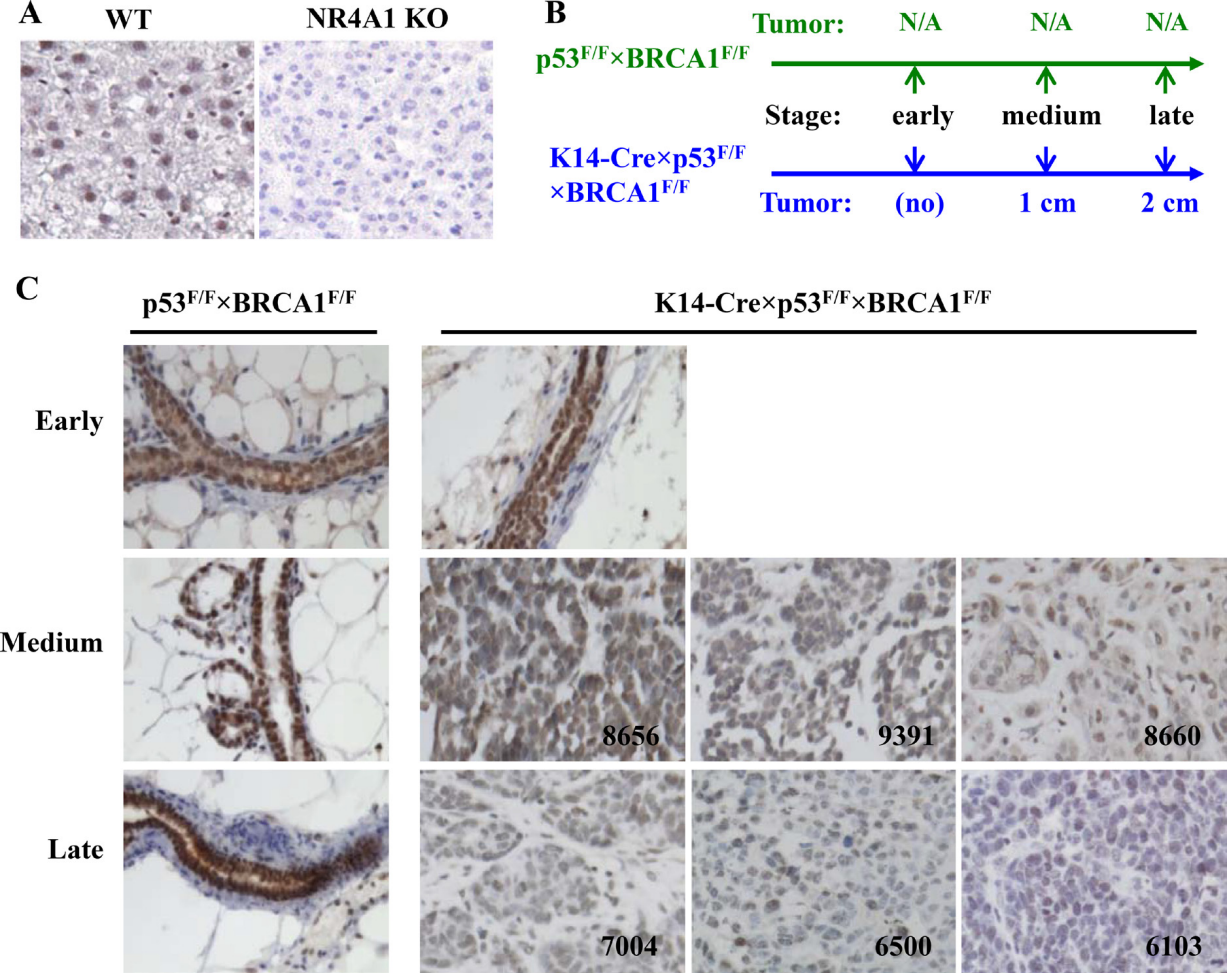
IHC analysis of NR4A1 protein expression in the mouse basal-like mammary gland tumors (**A**) Validation of NR4A1 antibody specificity by using the liver sections prepared from NR4A1 WT (positive control) and knockout (negative control) mice. (**B**) The schedules for collecting mouse mammary glands and tumors from p53^F/F^×BRCA1^F/F^ and K14-Cre×p53^F/F^×BRCA1^F/F^ mice. The early time point was at the mouse age of 4 months. The medium and late time points for K14-Cre×p53^F/F^×BRCA1^F/F^ mice were the time points when the diameters of their mammary tumors reached 1 and 2 cm, respectively, while these time points for p53^F/F^×BRCA1^F/F^ mice were the age points that matched each K14-Cre×p53^F/F^×BRCA1^F/F^ mouse with 1 or 2 cm tumor. (**C**) Analysis of NR4A1 in normal mammary glands of p53^F/F^×BRCA1^F/F^ mice and non-tumor mammary glands and different stage tumors of K14-Cre×p53^F/F^×BRCA1^F/F^ mice by IHC (brown color). The tumor ID numbers are indicated.

We first established the working condition of NR4A1 antibody for IHC by using liver sections prepared from WT mouse as a positive control and NR4A1 knockout mouse as a negative control [9]. The NR4A1 antibody detected NR4A1 protein located mainly in the nuclei of WT mouse liver cells but did not detect any signal in the knockout mouse liver cells (Figure 2A). This indicates that the NR4A1 antibody worked specifically.

Next, we used the previously established K14-Cre×p53^F/F^×BRCA1^F/F^ mice as a basal-like breast cancer model and p53^F/F^×BRCA1^F/F^ mice as a normal control to study NR4A1 expression changes during tumor growth and progression [21]. In female K14-Cre×p53^F/F^×BRCA1^F/F^ mice, the mammary tumorigenesis was induced by the deletion of both p53 and BRCA1 in the K14-expressing basal (myoepithelial) cells, and palpable tumors could be detected at ages of 6–7 months. The p53^F/F^×BRCA1^F/F^ mice had functional p53 and BRCA1 genes and did not develop any mammary tumors. High-level NR4A1 protein was mainly detected in the nuclei of the luminal and myoepithelial cells of the p53^F/F^×BRCA1^F/F^ mouse mammary glands at all examined stages (Figure 2B and 2C). At the age of 4 months when K14-Cre×p53^F/F^×BRCA1^F/F^ mice had not developed any mammary tumor (early stage), high-level NR4A1 protein was also detected in the mammary gland luminal and myoepithelial cells of these mice. However, when their tumor sizes grew to ˜1 cm in diameter (medium stage), NR4A1 protein in the nuclei of tumor cells was significantly reduced to medium to low levels in individual tumors. When their tumor sizes reached ˜2 cm in diameter (late stage), NR4A1 protein in individual tumors was further reduced to low or negative levels (Figure 2B and 2C). These results indicate that NR4A1 is progressively downregulated during the growth and progression of the spontaneously developed basal-like mouse mammary tumors.

### NR4A1 protein is decreased in human TNBC

**Figure 3:**
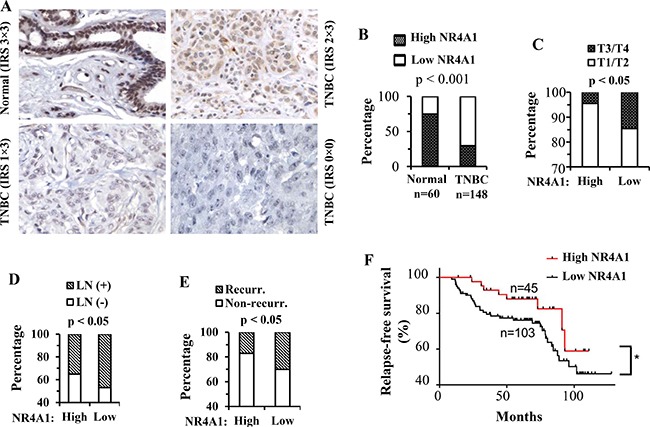
NR4A1 protein in human TNBC and its association with clinicopathological characteristics and disease recurrence (**A**) Representative images of NR4A1 IHC in human normal breast tissue and TNBC tumors. Immunoreactive score (IRS) was determined by multiplying the intensity score with the positive cell percentage score. (**B**) The percentages of high-NR4A1 (IRS: 6–9) and low-NR4A1 (IRS: 0–4) samples in normal and TNBC tumor groups. The tumor numbers and the *p* value calculated by Chi-squared test are indicated. (**C**–**E**) Percentages of T3/T4 and T1/T2 stage tumors, patients with and without lymph node (LN) metastasis, and patients with and without disease recurrence in high-NR4A1 and low-NR4A1 tumor groups. High-NR4A1 and low-NR4A1 groups had 45 and 103 cases, respectively. The indicated *p* values were calculated by Chi-squared test. (**F**) Kaplan Meier relapse-free survival curves for patients with the high-NR4A1 and low-NR4A1 TNBC tumors. *p < 0.05 by Gehan-Breslow-Wilcoxon test.

Next, we obtained tissue microarrays containing 60 normal human breast samples and 148 human TNBC samples with patient clinicopathologic data (Table 1). We performed NR4A1 IHC on these tissue microarrays and obtained NR4A1-immunoreactive score (IRS) from normal breast epithelial cells or TNBC cells in each sample. We found that 75% (45 out of 60) of the normal breast samples expressed high-level NR4A1 protein and only 25% (15 out of 60) of these samples had low-level NR4A1 protein. However, only 30% (45 out of 148) of TNBC samples had high-level NR4A1, and the other 70% (103 out of 148) of these samples showed low-level NR4A1 (Figure 3A and 3B). Statistical analysis of these data revealed a significant decrease in NR4A1 protein expression in TMBC samples versus normal breast samples.

**Table 1:** Clinicopathologic features of TNBC patients

Total number of patient samples	148
Number of patients with age data Age distribution (year): Min (28) Median (51) Mean (52) Max age (81)	97
Patients without age data	51
Tumor stage	
T1 and T2	131
T3 and T4	17
Axillary lymph node status	
Negative	85
Positive	63
Recurrence	
Yes	39
No	109
Survival	
Yes	118
No	30

### Low NR4A1 protein expression in TNBC tumors is associated with advanced tumor stages, lymph node metastasis and poor relapse-free survival

The TNBC patients in this study were divided into high NR4A1 (*n* = 45) and low NR4A1 (*n* = 103) TNBC groups. Several clinicopathological characteristics including tumor stage, lymph node metastasis, recurrence rate, relapse-free survival time and overall survival time were statistically compared between these two groups. Firstly, only 4% of the high-NR4A1 tumors were at T3 or T4 stages, while as many as 15% of the low-NR4A1 tumors were at T3 or T4 stages (Figure 3C), suggesting that decreased NR4A1 is associated with advanced TNBC stages. Secondly, only 35% of the patients with high-NR4A1 tumors had lymph node metastasis, but more than 48% of the patients with low-NR4A1 tumors developed lymph node metastasis (Figure 3D), suggesting that decreased NR4A1 is associated with lymph node metastasis in TNBC patients. Thirdly, after the initial surgical therapy, about 17% of the patients with high-NR4A1 tumors showed disease recurrence, while as many as 30% of the patients with low-NR4A1 tumors had disease recurrence (Figure 3E). Kaplan-Meier relapse-free survival analysis revealed that the patients with low-NR4A1 tumors had a significantly worse relapse-free survival time than the patients with high-NR4A1 tumors (Figure 3F). Finally, the patients with low NR4A1 tumors showed an obvious trend of worse overall survival than the patients with high NR4A1 tumors (data now shown), but the difference in overall survival time between these two patient groups did not reach a statistically significant level (*p* = 0.1285 by Gehan-Breslow-Wilcoxon test). Together, these results suggest that decreased NR4A1 protein in TNBC tumors is associated with increased TNBC progression including more advanced tumor stages, higher metastasis frequency, more recurrence incidence and poorer relapse-free survival.

### Overexpression of NR4A1 inhibits the proliferation, viability, motility and invasiveness of MDA-MB-231 TNBC cells

**Figure 4:**
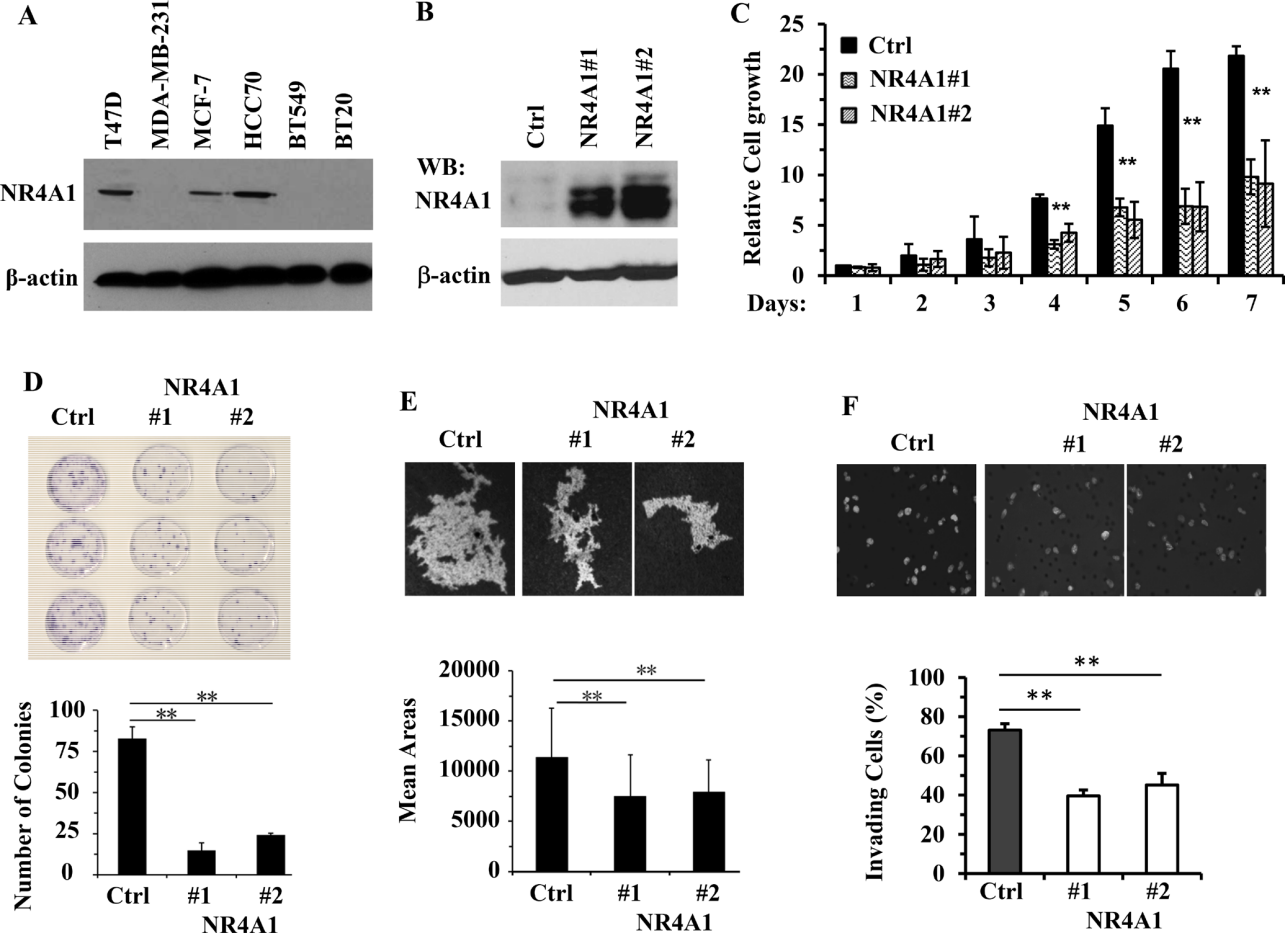
NR4A1 expression inhibits the growth, viability, migration and invasion of MDA-MB-231 cells (**A**) Immunoblotting analysis of NR4A1 in the indicated breast cancer cell lines. (**B**) Immunoblotting analysis of the stable MDA-MB-231 cell pool bearing an empty retroviral vector (Ctrl) or the two cell lines with retrovirus-mediated stable expression of NR4A1. (**C**) Cell growth rates determined by MTS assay. The indicated cells were cultured overnight, followed by a MTS assay to determine the baseline OD value. Cells were cultured for 7 more days and MTS assay was performed daily. The relative cell growth was presented as fold increases of the OD values over the baseline OD values for each group. Data were collected from 6 assays. (**D**) Representative images of the colonies derived from 231-Ctrl, 231-NR4A1#1 and 231-NR4A1#2 cells in a low cell density setting, and the average number of colonies formed from each cell group. Experiments were performed with three repeats. (**E**) Representative trail images swept by individual 231-Ctrl, 231-NR4A1#1 and 231-NR4A1#2 cells on the fluorescent bead-covered culture plates. The mean trail area (pixels) swept by each cell was obtained from measuring migration trail areas of 45 individual cells. (**F**) Representative images of 231-Ctrl, 231-NR4A1#1 and 231-NR4A1#2 cells that invaded through the Matrigel layer in the transwell invasion assay. The nuclei of invaded cells attached to the lower side of the membrane was stained by DAPI and imaged at 200× magnification under a fluorescence microscope. Cell migration was separately measured in parallel using the transwell apparatus without Matrigel (images not shown). The invasion index was the percent of invaded cell number to the migrated cell number as described in Materials and Methods. The assay was performed with 4 repeats. Data in panels (C–F) are presented as Mean ± SEM. **p < 0.01 by One-Way ANOVA test.

Our data demonstrating the reduction of NR4A1 in TNBC tumors allowed us to propose that NR4A1 expression might be negatively correlated with the malignancy of TNBC cells. To choose a TNBC cell line for studying the role of NR4A1 expression, we examined NR4A1 protein in several breast cancer cell lines. The ERα-positive breast cancer cells T47D and MCF-7 expressed high NR4A1 protein, while only one of the four examined TNBC cell lines, HCC70, showed NR4A1 protein expression. The other three TNBC cells, MDA-MB-231, BT549 and BT20, did not express NR4A1 protein (Figure 4A). To test the effect of restored NR4A1 expression on the malignant features of TNBC, we first generated two stable MDA-MD-231 cell lines with retrovirus-mediated NR4A1 expression (hereafter, designated as 231-NR4A1#1 and 231-NR4A1#2 cells) and their control cells transduced by the empty retrovirus (hereafter, designated as 231-Ctrl cells) (Figure 4B). We then compared these cells with different NR4A1 expression levels for their growth rates by MTS cell viability assay, survival ability by colony-forming assay, motility by tracing individual cell migration, and invasiveness by trans-well assay. We found that the growth rates of 231-NR4A1#1 and 231-NR4A1#2 cells were reduced more than 2 fold compared with 231-Ctrl cells after one week in culture (Figure 4C). The number of colonies formed from 231-NR4A1#1 and 231-NR4A1#2 cells were 4–5 times lesser than the number of colonies formed from 231-Ctrl cells. The colonies of 231-NR4A1#1 and 231-NR4A1#2 cells also were much smaller in size and contained markedly less number of cells than those formed from 231-Ctrl cells (Figure 4D). 231-NR4A1#1 and 231-NR4A1#2 cells also showed significantly decreased capabilities to migrate on the culture plate and to invade through a Matrigel layer as compared to 231-Ctrl cells (Figure 4E and 4F). These results indicate that NR4A1 expression in MDA-MB-231 TNBC cells could effectively inhibit cell proliferation, viability, motility and invasiveness.

### NR4A1 downregulates the JNK/AP-1/cyclin D1 pathway and attenuates the G1 phase of cell cycle

**Figure 5:**
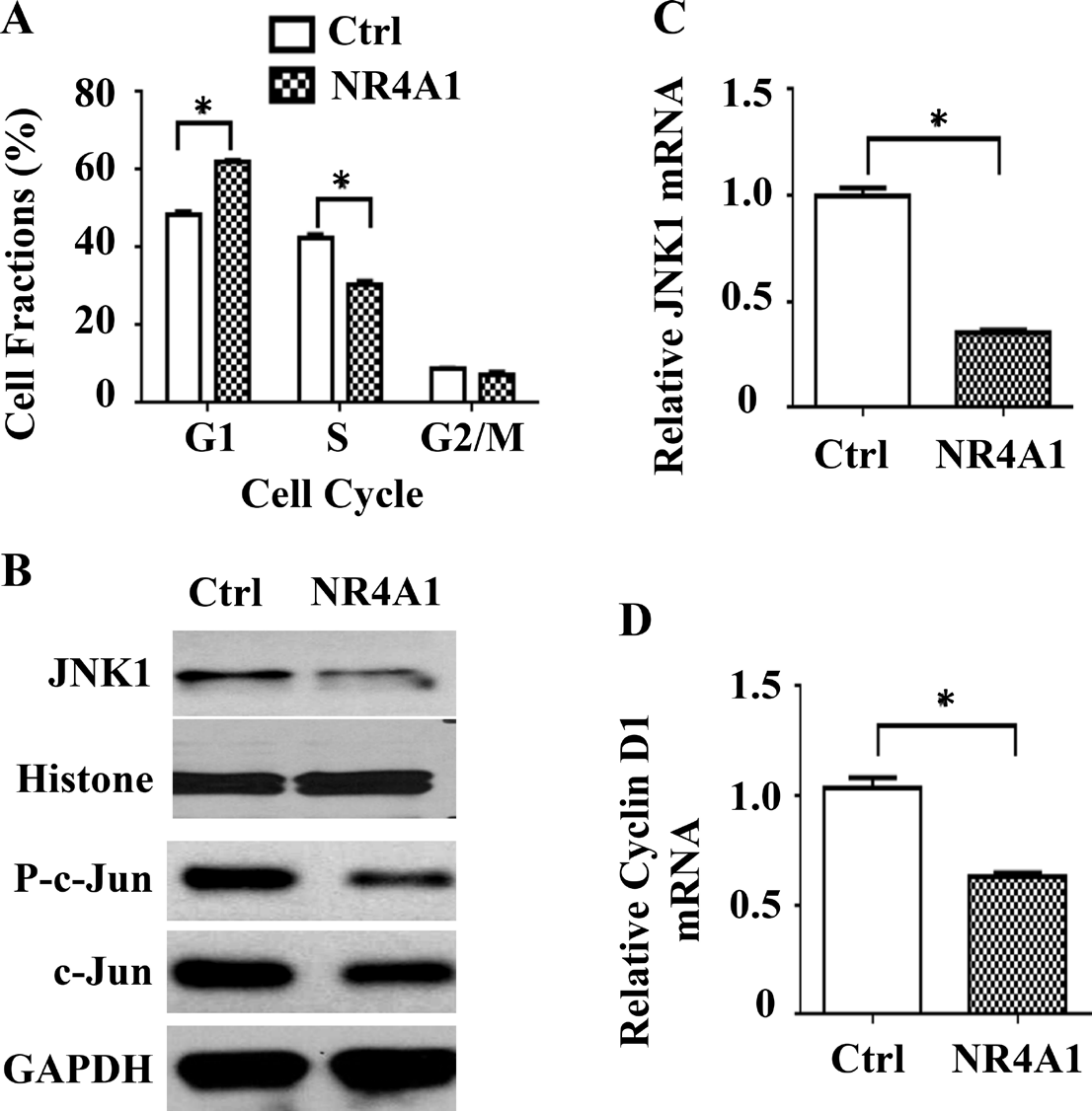
The role of NR4A1 in cell cycle regulation (**A**) Cell cycle analysis. DAPI-stained 231-Ctrl and 231-NR4A1 cells were analyzed by flow cytometry. The average percentages of cell fractions in G1, S and G2/M phases were obtained from three assays. (**B**) Immunoblotting analysis of JNK, phosphorylated c-Jun and total c-Jun in 231-Ctrl and 231-NR4A1 cells. Histone or GAPDH were served as loading controls. (**C** and **D**) qPCR analysis of JNK and cyclin D1 mRNA expression in 231-Ctrl and 231-NR4A1 cells. The relative expression levels of JNK and cyclin D1 were normalized to the expression level of endogenous β-actin mRNA. Assays were performed in triplicates. Data are presented as mean ± SEM. *p < 0.05 by two-tailed Student's *t* test.

To characterize the feature of NR4A1-inhibited cell proliferation, we performed cell cycle analysis. We found that 50.60% and 39.44% of 231-Ctrl cells were in the G1 and S phases, respectively. However, the 231-NR4A1 cells had as many as 57.34% in G1 phase and as few as 32.96% in S phase, which were significantly different from the 231-Ctrl cells (Figure 5A). These results indicate that NR4A1 overexpression results in a G1 arresting in the MDA-MB-231 TNBC cells.

Since growth factors usually activate the MAPK and/or AKT pathways to stimulate G1 phase of the cell cycle, we examined the expression levels of total and active forms of MAPKs including ERK, p38 and JNK as well as AKT by Western blotting. We found that neither total nor active forms of ERK1/2, p38 and AKT showed any difference between 231-Ctrl and 231-NR4A1 cells. However, the total JNK protein was significantly reduced in 231-NR4A1 cells versus 231-Ctrl cells (Figure 5B). Quantitative RT-PCR analysis also revealed that JNK mRNA was downregulated in 231-NR4A1 cells versus 231-Ctrl cells, suggesting that NR4A1 expression could suppress the expression of JNK at the transcriptional level (Figure 5C). Due to a failure to detect a clear band of the active form of JNK protein in our cell lysates, we turned to determine JNK activity by assaying the phosphorylation degree of c-Jun, a known substrate of JNK [22, 23]. We found that the JNK-mediated phosphorylation of c-Jun was drastically reduced although the total c-Jun protein was also slightly reduced in 231-NR4A1 cells versus 231-Ctrl cells (Figure 5B). These results demonstrate that NR4A1 expression down-regulates JNK expression and attenuates the JNK/AP-1 signaling pathway in MDA-MB-231 cells.

Since the JNK1–AP-1 pathway promotes cyclin D1 expression and cyclin D1 drives cell cycle by accelerating G1 phase [22, 23], we next examined cyclin D1 expression. Indeed, cyclin D1 expression is significantly reduced in 231-NR4A1 cells versus 231-Ctrl cells (Figure 5D). These results suggest that NR4A1 expression can attenuate the activity of the JNK1–AP-1–cyclin D1 pathway to inhibit G1 phase progression of the cell cycle in TNBC cells.

### NR4A1 expression inhibits the growth and metastasis of MDA-MB-231 cell-derived tumors in mice

**Figure 6:**
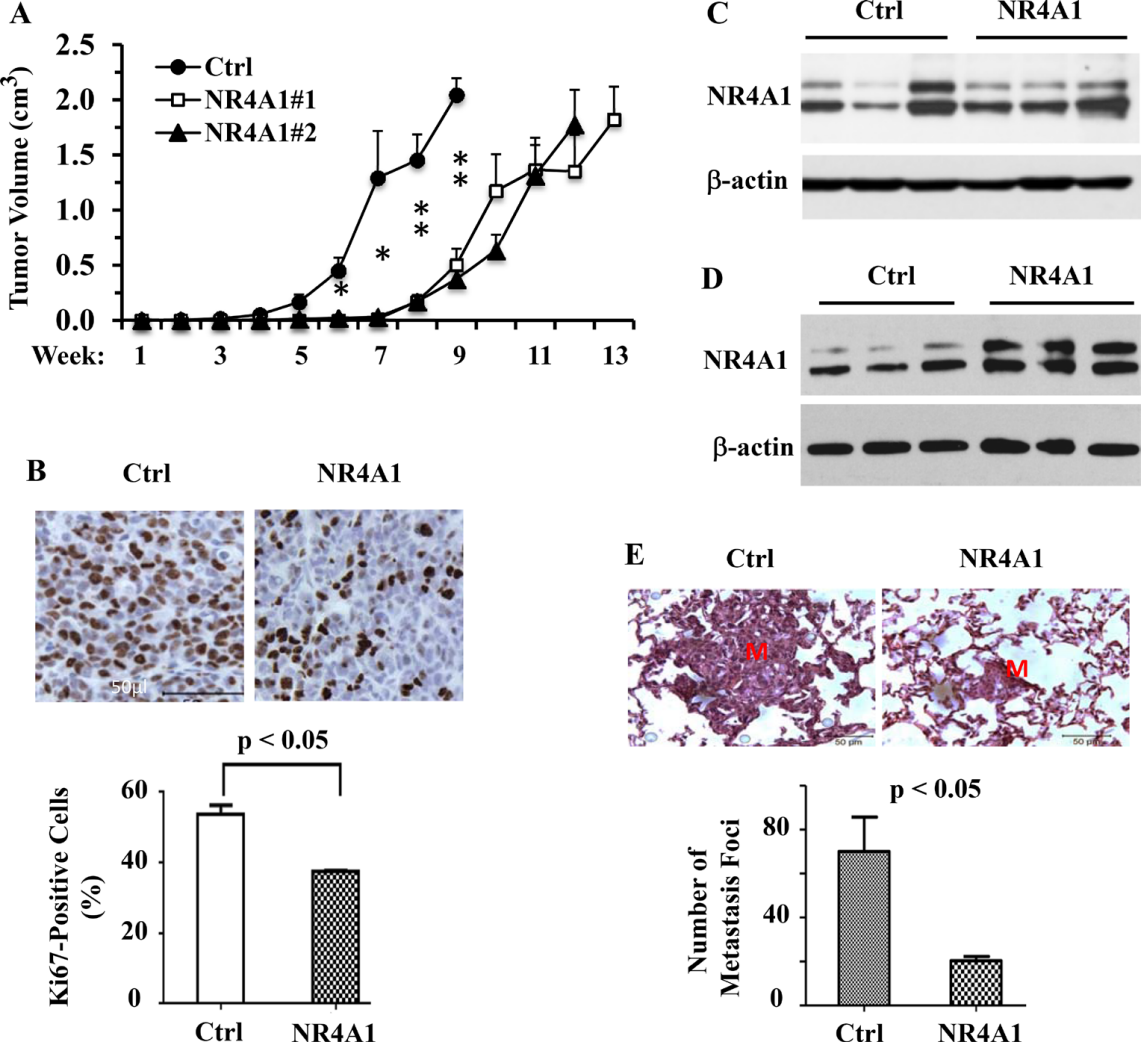
NR4A1 expression inhibits tumor growth and suppresses lung metastasis in SCID mice (**A**) The growth curves of 231-Ctrl, 231-NR4A1#1 and 231-NR4A1#2 cell-derived xenograft tumors in 12, 8 and 8 SCID mice, respectively. Tumor volume was measured once a week as indicated. * and **p < 0.05 and 0.01, respectively. (**B**) Ki67 IHC staining on the tissue sections prepared from the 231-Ctrl and 231-NR4A1#1 cell-derived xenograft tumors. The average percentage of Ki67-positive tumor cells was obtained from four individual tumors in each group. (**C**) Immunoblotting analysis of NR4A1 expression in the late-stage 231-Ctrl (*n* = 3) and 231-NR4A1#1 (*n* = 3) xenograft tumors shown in panel A. β-actin served as a loading control. (**D**) Immunoblotting analysis of NR4A1 expression in the early-stage 231-Ctrl (*n* = 3) and 231-NR4A1#1 (*n* = 3) xenograft tumors harvested in 4 weeks after cells were injected into SCID mice. β-actin served as a loading control. (**E**) Analysis of lung metastasis in SCID mice (*n* = 6 in each group) with 231-Ctrl and 231-NR4A1#1 xenograft tumors shown in panel A. The representative images of the H&E-stained lung sections contained a large and a small metastasis nodules in mice with 231-Ctrl or 231-NR4A1#1 cell-derived xenograft tumors as indicated. “M” indicates the metastasis nodule. The average number of metastasis nodules per mouse lung were obtained by counting the metastasis foci observed on the lungs of mice with 231-Ctrl or 231-NR4A1 tumors shown in panel A under a dissection stereo microscope.

To assess the tumor suppressor function of NR4A1 in the inhibition of TNBC growth *in vivo*, we injected 231-Ctrl, 231-NR4A1#1 and 231-NR4A1#2 cells into the mammary gland fat pads of female SCID mice and measured their tumor growth rates. We found that the tumors derived from 231-Ctrl cells became palpable 4 weeks after injection. However, the tumors derived from 231-NR4A1#1 and 231-NR4A1#2 cells were not observed until 8 weeks after injection. Furthermore, the growth rates of the 231-NR4A1#1 and 231-NR4A1#2 tumors were slower than those of the 231-Ctrl tumors in the early stage after becoming palpable (Figure 6A). IHC staining of Ki-67, a cell proliferation marker, revealed that the average cell proliferation rate of 231-NR4A1 tumors was significantly reduced (Figure 6B). These results demonstrate that NR4A1 plays an important role to inhibit the initiation and growth of the MDA-MB-231 cell-derived tumors in mice by reducing their proliferation rate.

Although the 231-NR4A1 tumors took much longer time to become palpable and initially grew slower than 231-Ctrl tumors, the growth rates (the slope of the tumor growth curves) of the 231-NR4A1 tumors gradually became less different to that of the 231-Ctrl tumors (Figure 6A). To search for an explanation, we examined NR4A1 protein by Western blotting in 231-Ctrl and 231-NR4A1 tumors that were respectively harvested at weeks 9 and 12 when tumor sizes in both groups became comparable (Figure 6A). Surprisingly, NR4A1 protein was detected at similar levels in both 231-Ctrl and 231-NR4A1 tumors (Figure 6C), suggesting that the much higher level of NR4A1 protein in 231-NR4A1 cells versus 231-Ctrl cells failed to maintain during the growth and progression period in the 231-NR4A1 tumor cells. To support this notion, we injected 231-Ctrl and 231-NR4A1 cells into the mammary gland fat pads of SCID mice and harvested the very small tumors formed from these cells at a week-4 early time point and examined NR4A1 in these small tumors. We found that NR4A1 protein was much higher in the 231-NR4A1 tumors versus 231-Ctrl tumors at this early stage (Figure 6D). In addition, low and moderate levels of NR4A1 protein was detected in the early stage 231-Ctrl tumors and the late stage 231-Ctrl and 231-NR4A1 tumors, which could be contributed from the stromal and immune cells in the tumor microenvironment. These observations suggest that some of the 231-NR4A1 cells may lose NR4A1 overexpression and became a dominant subpopulation in the late stage tumors due to their growth advantage over those cells expressing high level NR4A1.

We also examined lung, liver and mesenteric lymph nodes for distant metastasis in SCID mice bearing 231-Ctrl and 231-NR4A1 cell-derived xenograft tumors at the experimental end points when tumor sizes became comparable (Figure 6A). We found lung metastasis but no visible metastasis in other organs. The average number of metastatic foci observed in the lungs of mice with 231-NR4A1 tumors was markedly reduced when compared with that in mice with 231-Ctrl tumors. The metastatic foci derived from 231-NR4A1 tumors were also much smaller than that derived from 231-Ctrl tumors as examined in the H&E-stained lung sections (Figure 6E). These results demonstrate that NR4A1 expression can significantly decrease the metastatic capability of MDA-MB-231 cell-derived tumors *in vivo*.

## DISCUSSION

In this study, we performed bioinformatic analysis of large data sets, which revealed a decrease in NR4A1 mRNA expression in human TNBC. We found a progressive downregulation of NR4A1 protein in the basal-like mammary gland tumors developed in K14-Cre×p53^F/F^×BRCA1^F/F^ mice. We also performed semi-quantitative analysis of NR4A1 protein expression in human normal breast epithelium and TNBC samples and found that NR4A1 protein is frequently downregulated in TNBC. These multiple lines of evidence clearly indicate that NR4A1 expression is frequently decreased in TNBC. More importantly, we found that the decreased levels of NR4A1 expression in TNBC are associated with advanced tumor stages, lymph node metastasis and worse relapse-free survival. These findings suggest that the decreased NR4A1 expression may play a role in promoting TNBC progression and serve as an indicator of worse prognosis.

Our data demonstrated that NR4A1 protein is expressed in T47D and MCF-7 cells, which are ERα-positive breast cancer cells, but is barely detected in three out of four TNBC cell lines including MDA-MB-231, BT549 and BT20 cells. Interestingly, restored expression of NR4A1 in MDA-MB-231 cells significantly decreased the proliferation, surviving capability, mobility and invasiveness of these cells in culture. In SCID mice, the xenograft tumors derived from 231-NR4A1 cells with NR4A1 expression showed much slower growth rates and developed much lesser lung metastasis than the tumors derived from 231-Ctrl cells without ectopic NR4A1 expression. These results indicate that NR4A1 expression can significantly suppress the aggressive features of MDA-MB-231 TNBC cells in both culture and *in vivo* conditions. Intriguingly, the implanted 231-NR4A1 cells took a much longer latency than 231-Ctrl cells did to develop palpable tumors, but the 231-NR4A1 tumors appeared to gradually increase their growth rates at a later stage. Our further analysis revealed that the early stage 231-NR4A1 tumors showed much higher NR4A1 expression than the early stage 231-Ctrl tumors. This difference in NR4A1 expression should be responsible for the long latency and arrested growth of the 231-NR4A1 tumors at early stages. Interestingly, NR4A1 protein in the later stage 231-NR4A1 tumors was decreased to the similar levels as that in the later stage 231-Ctrl tumors, suggesting that the high NR4A1 expression was lost in most of the 231-NR4A1 tumor cells. This could be a consequence of growth selection for the tumor cells with low NR4A1 expression during the tumor progression process in mice. In addition, we noticed that NR4A1 protein was much higher in the 231-Ctrl tumors in mice versus the 231-Ctrl cells in culture. Since the tumors contain many other types of non-tumor cells such as fibroblast, vascular and immune cells, the NR4A1 detected in the 231-Ctrl tumors may be mainly contributed from these non-tumor cells although we cannot exclude the possibility that endogenous NR4A1 expression might be induced in 231-Ctrl tumor cells in the tumor environment.

It is well established that JNKs play an important role in cell apoptosis and tumor suppression [24, 25]. However, JNKs, especially JNK1, can also be a driver of cell proliferation and transformation in certain types of non-cancer cells and in those cancer cells with impaired cell apoptosis pathways [26]. It has been shown that the JNK-mediated phosphorylation of AP-1 (c-Jun) upregulates cyclin D1 expression to drive proliferation in liver cell regeneration and mouse epidermal cell transformation [22, 23]. In the present study, the tumor suppressor p53 is mutated in MDA-MB-231 cells [27] and thus, decreased JNK1 activity should attenuate the activity of the JNK1–AP-1–cyclin D1 signaling pathway, resulting in decreased cell proliferation and tumor growth. Indeed, we found that expression of NR4A1 in MDA-MB-231 cells inhibited JNK1 expression, c-Jun activation and cyclin D1 expression, which is correlated with the decreased cell proliferation and tumor growth of these cells. These results suggest that NR4A1-inhibited G1 arresting and cell proliferation are caused, at least in part, by the attenuated activity of the JNK1–AP-1–cyclin D1 pathway.

NR4A1 has been paradoxically reported as both a tumor suppressor and a cancer driver in previous studies, depending on different types of cancers, different cell lines of the same cancer type or different cohorts of patients [9–18, 28]. Since NR4A1 plays a role to induce apoptosis through inhibiting Bcl-2 [29, 30], the tumor suppressor role may be partially attributed to its function to mediating apoptosis. On the other hand, our study may partially elucidate one of the NR4A1 tumor suppressive roles in TNBC. Most BRCA1 and BRCA2 mutation-induced breast cancers are TNBC, and most (˜75%) TNBC contains mutated p53 [29–31]. Therefore, the cell apoptosis pathways could be impaired in a large proportion of TNBC. Our findings showing that NR4A1 controls JNK1 activity in MDA-MB-231 cells with mutated p53 support the notion that NR4A1 loss would promote TNBC growth and progression. On the other hand, one study reported that NR4A1 expression was increased in late stage breast cancer and this increase was associated with reduced overall survival [18]. This observation is opposite from ours, which could be due to distinct subtypes of breast tumors studied. All of our samples are TNBC, while it is unknown what subtypes of breast tumors were analyzed in this previous study [18]. The same study also showed that in MDA-MB-231 cells, NR4A1 knockdown decreased cell motility and NR4A1 overexpression increased bone metastasis [18]. These results are also different from ours observed from our experimental systems. MDA-MB-231 cells express undetectable endogenous NR4A1 protein under normal culture condition, so we did not perform migration or invasion assays using MDA-MB-231 cells with NR4A1 knockdown. Instead, we only overexpressed NR4A1 in these cells and observed a significant inhibition of cell motility by NR4A1 overexpression. The different results on metastasis could be due to the use of different model systems. The previous study performed intracardiac injection to preferentially direct cells to the bone, while our study injected cells into the mammary fat pads and then examined lung metastasis derived from the primary tumors in the mammary fat pads. In addition to the differences in the local invasion and intravasation steps, the same cancer cells may also have different capability to seed and grow in different organ sites such as the lung versus the bone. Therefore, these different results may reflect the complexity of TNBC, which requires further in depth analysis in the future studies.

When comparing the tumor formation latency and tumor growth profiles of 231-Ctrl and 231-NR4A1 cells in mice, we found that 231-NR4A1 tumors took a much longer latency to become palpable, grew slower in early phase, and then grew as fast as 231-Ctrl tumors in later phase. Interestingly, we found that this growth profile negatively correlated with NR4A1 protein levels in the tumors. Most likely, in early phase, the high level NR4A1 suppressed tumor formation and growth. In late phase, some of the tumor cells might lose high NR4A1 expression, escape from its suppression, and acquire growth advantage to become dominant subpopulations in big tumors. On the other hand, we also could not exclude another possibility that NR4A1 protein might be induced in 231-Ctrl tumor cells in the tumor environment. However, since NR4A1 protein level did not change much in the early and late phase 231-Ctrl tumors and the tumors also contain many other cell types from the host mice such as fibroblasts, immune cells and vascular cells, it is more likely that the NR4A1 protein detected in 231-Ctrl tumors might be from those non-tumor cells in the tumor environment.

A unique feature of most NRs is to serve as a ligand-dependent transcription factor to regulate physiological functions or control pathological processes. Based on this feature, many agonists or antagonists have been developed to treat a variety of human diseases. Specific to NR4A1, although its physiological hormone or ligand has not been identified, cytosporone B has been reported to be a small molecular compound to bind and activate NR4A1. This ligand-receptor interaction is also reported to induce apoptosis and inhibit tumor growth of colon cancer cells [32]. However, in our tests both the 231-Ctrl cells with little endogenous NR4A1 and the 231-NR4A1 cells with NR4A1 overexpression showed little response to cytosporone B treatment and no treated cells were apoptotic. This suggests that either this compound did not activate NR4A1 or NR4A1 activation was unable to induce apoptosis in these cells with mutant p53. It will be important to test the effects of more endogenous and synthetic agonists and antagonists of NR4A1 on the growth and metastasis of TNBC cells in the future studies once these NR4A1 ligands become available.

## MATERIALS AND METHODS

### Mouse models and mouse tissue sampling

Animal protocols were approved by the Institutional Animal Care and Use Committee at Baylor College of Medicine. NR4A1 knockout (KO) mice and K14-Cre×p53^F/F^×Brca1^F/F^ mice were generated and characterized as described previously [9, 21]. The liver tissues were isolated from adult NR4A1 KO and age-matched wild type (WT) female mice. The K14-Cre×p53^F/F^×Brca1^F/F^ founder mice with a FVB strain background were first bred with WT FVB mice to generate K14-Cre×p53^F/+^×Brca1^F/+^ and p53^F/+^×Brca1^F/+^ mice. Then, these heterozygous mice were further crossbred to produce K14-Cre×p53^F/F^×Brca1^F/F^ and p53^F/F^×Brca1^F/F^ female mice for experiments. Double deletion of both floxed *p53* and *Brca1* genes in the K14-expressing myoepithelial (basal) cells results in spontaneous development of basal-like mammary tumors in K14-Cre×p53^F/F^×Brca1^F/F^ mice during 6–7 months of age [21]. The mammary glands were isolated from 4-month-old K14-Cre×p53^F/F^×Brca1^F/F^ mice, and tumors were isolated from these mice when the diameters of individual tumors reached 1 or 2 cm. The normal control mammary glands were isolated from age-matched p53^F/F^×Brca1^F/F^ mice. Each group had at least five mice at each time point. Collected mouse tissues were fixed overnight in 4% paraformaldehyde in phosphate-buffered saline (PBS) at 4°C, washed with PBS, dehydrated in ethanol, embedded in paraffin and cut into 5-μm-thick sections.

### Nr4a1 immunohistochemistry (IHC) in mouse tissues

IHC was performed as described previously [33, 24]. Briefly, mouse liver, mammary gland and tumor tissue sections were deparaffinized in xylene and rehydrated by going through ethanol series and water. Antigen retrieval was conducted in 10 mM sodium citrate (pH 6.0) by heating in a high-pressure cooker for 2 minutes. The sections were washed in PBS and blocked with PBS containing 10% fetal bovine serum (FBS) for 1 hour at room temperature. The prepared tissue sections were incubated overnight at 4°C with polyclonal NR4A1 antibody (LS-B114, Lifespan Bioscience, Seattle, WA) at 1:500 dilutions in PBS with 10% horse serum. The secondary antibody was anti-rabbit IgG conjugated with horseradish peroxydase (HRP). The signal was visualized using DAB kit. The sections were counterstained with Harris Modified Hematoxylin and mounted with Permount. Immunostained sections were examined under microscope and imaged by a real-time CCD camera under identical conditions.

### NR4A1 IHC in human breast and TNBC samples

Human normal breast and TNBC tumor tissue microarrays were constructed in Department of Pathology at University of Texas (UT) Southwestern Medical Center, Department of Pathology at UT MD Anderson Cancer Center and The First Affiliated Hospital at Sun Yat-Sen University. In this study, tissue microarrays containing 60 normal human breast tissues and 148 TNBC samples were used for NR4A1 IHC. IHC was performed as described above for mouse mammary gland and tumor sections. NR4A1 immunoreactivity scores (IRS) were evaluated by two pathologists (J.B & Z.Y.) and an investigator (H.W.) according to a previously established semi-quantitative system [35]. In this scoring system, the immunoreactivity intensity was scored on a scale of 0 (negative), 1 (weak), 2 (moderate) and 3 (strong), and the percent of positively stained tumor cells was scored on a scale of 0 (< 10%), 1 (10–30%), 2 (30–60%) and 3 (> 60%). Then, the combined score (0–9) for each sample was obtained by multiplying these two category scores. Samples with less than 50 tumor cells were excluded from data analysis.

### Data analysis of the clinicopathological correlations with NR4A1 IRSs

All samples with IHC staining data were divided into high-NR4A1 (IRSs = 6–9) and low-NR4A1 (IRSs = 0–4) groups. Pearson's Chi-square test was used to compare NR4A1 expression in normal tissue and TNBC, and to analyze the association between NR4A1 expression in TNBC patients and clinicopathological characteristics including tumor stage (T1 and T2 vs. T3 and T4), lymph node metastasis status (positive vs. negative), disease recurrence (yes vs. no) and overall survival (yes vs. no). Patient relapse-free survival or disease recurrence was defined as the time in months from the clinical data of diagnosis to the first recurrence of the disease. Patients’ TNBC relapse-free survival was calculated using the Kaplan-Meier method and compared using the Gehan-Breslow-Wilcoxon test. A *p*-value of less than 0.05 was considered to be significant.

### Cell culture and generation of NR4A1-expressing cell lines

T47D and BT549 human breast cancer cells were cultured in RMPI-1640 medium with 10% FBS in a 37°C humidified incubator supplemented with 5% CO_2_ and 95% air. MDA-MB-231, HCC70, MCF-7 and BT-20 human breast cancer cells were cultured in DMEM medium with 10% FBS in an incubator with same conditions. 293T cells were transfected with pRetroX-TRE retroviral empty plasmids (Clontech, Mountain View, CA) or pRetroX-TRE-NR4A1 retroviral plasmids together with pCMV-Gag-Pol and pAmpho plasmids for expressing retroviral-packaging proteins by using Lipofectamine 2000 reagent (Invitrogen, Carlsbad, CA). The generated retrovirus was used to infect MDA-MB-231 cells and the infected cells were selected in medium containing hygromycin B for 7 days. The surviving cells infected by the retrovirus without NR4A1 expression cassette were designated as 231-Ctrl cells, which were used as control cells in all experiments. The surviving cell colonies infected by the retrovirus with NR4A1 expression cassette were individually isolated, expanded and examined for NR4A1 protein expression by Western blotting. Two clones, 231-NR4a1#1 and 231-NR4A1#2, with stable NR4A1 protein expression were established for experimental analysis.

### Immunoblotting

Immunoblotting was carried out as described previously [36, 37]. Briefly, cells were washed with PBS and lysed in RIPA buffer containing 1.0% Triton X-100, 0.5% sodium deoxycholate, 0.1% SDS, 150 mM NaCl, 50 mM Tris–HCl (pH 8.0), 10 mg/ml pepstatin A, 10 mg/ml leupeptin and 1 mM phenylmethylsulfonyl fluoride (PMSF). After centrifuging for 10 minutes at 4°C, supernatant with 20 mg protein for each sample was subjected to immunoblotting analysis. Primary antibodies used for immunoblotting were against NR4A1 (#3960), JNK1 (#9258), c-Jun (#9165) and phosphorylated c-Jun (P-c-Jun) (#3270) (Cell Signaling, Danvers, MA).

### Cell proliferation and cell cycle assays

Cell growth assay was performed using an MTS-based CellTiter 96 AQueous One Solution Cell Proliferation Assay kit (Promega, Madison, WI). Briefly, 231-Ctrl, 231-NR4A1#1 and 231-NR4A1#2 cells were seeded in 96-well plate at a density of 2000 cells/well with 0.1 ml of DMEM medium containing 10% FBS. After 12 hours, MTS assay was performed according to the manufacturer's protocol to obtain baseline data at starting time. Specifically, 20 μl of CellTiter 96R AQueous One Solution were added to each well, the plates were incubated at 37°C for 3 hours, and the OD values were measured at a wavelength of 490 nm. Then, MTS assays were performed once a day until day 7. Relative cell growth data were calculated by normalizing to the baseline data at starting time. For cell cycle analysis, cells were washed in PBS, fixed in 70% ethanol for 2 hours, and stained with 4’,6-diamidino-2-phenylindole (DAPI). Stained cells were measured by flow cytometry using a LSRII Cell Analyzers and cell cycle data were calculated by using the FlowJo Version 7.6.5 software.

### Colony formation assay

231-Ctrl, 231-NR4A1#1 and 231-NR4A1#2 cells were seeded at a density of 100 cells per 10-cm culture plate in triplicates with DMEM medium containing 10% FBS and allowed to grow for 3 weeks. The formed cell colonies were stained with 0.05% crystal violet for 20 minutes and washed with PBS. Each plate with the stained colonies was imaged and the number of colonies formed in each plate was counted under a stereomicroscope.

### Cell migration assays

Cell migration was individually measured by using the Cellomics^®^ Cell Motility kit (Thermo Scientific, Waltham, MA). The blue fluorescent beads were added to a fibronectin-coated 96-well plate (1.5 μg/well). The plate was incubated for one hour at 37°C in dark and then washed 5 times to remove any extra beads. 231-Ctrl, 231-NR4A1#1 and 231-NR4A1#2 cells were suspended in DMEM medium with 10% FBS at a density of 400 cells/ml. Fifty μl of cell suspension with ˜20 cells was gently added to each well. After adding another 50 μl of serum-free DMEM medium, the cells were cultured for 18 hours. Then, the cells were fixed in 5.5% formaldehyde for 1 hour, permeabilized, and stained with Rhodamine-Phalloidin by following the kit protocol. After washing 3 times and adding 200 μl of washing buffer to each well, the plate was examined, and the migrating trails mapped by individual cell migration were imaged under a fluorescent microscope at 400×. For each well, 5–10 images were recorded. The areas of individual cell-mapped trails were quantitatively analyzed using the NIH Image J software. About 40–50 trails made by single cells were measured for each group.

### Cell invasion assay

Cell invasion assay was performed as described previously [37]. Fifty thousands of 231-Ctrl, 231-NR4A1#1 or 231-NR4A1#2 cells in 200 μl of serum-free DMEM medium were seeded in the upper chambers of the 24-well Transwell plate (BD Biosciences, San Jose, CA) with or without a pre-coated Matrigel layer. The lower chambers were filled with DMEM medium with 5% FBS. After culturing for 20 hours, cells remained in the upper chambers were removed and cells that migrated or invaded through the membrane from the upper chambers to the lower chambers were stained with DAPI, imaged under a fluorescent microscope and counted. The invasion index was obtained by normalizing the number of invaded cells to the number of migrated cells by following the manufacturer's instructions.

### RNA isolation and quantitative RT-PCR (qPCR)

Total RNA was isolated from 231-Ctrl and 231-NR4A1 cells by using Trizol reagent (Invitrogen, Carlsbad, CA). One μg of RNA from each sample was reversely transcribed into cDNA using a Reverse Transcription Kit (Eurogentec, Fremont, CA). TaqMan qPCR was performed with human JNK1 or cyclin D1 mRNA-specific primers and matched probes from the Universal Fluorescence-labeled Probe Library (Roche, Basel, Switzerland). For measuring JNK1 mRNA, the forward primer 5′-gggcagccctctccttta, the reverse primer 5′-cattgacagacgacgatgatg and the #89 probe were used. For measuring cyclin D1 mRNA, the forward primer 5′-ccatccagtggaggtttgtc, the reverse primer 5′-gtgggacaggtggccttt and the #20 probe were used.

### Xenograft tumor growth and metastasis in SCID mice

The 231-Ctrl, 231-NR4A1#1 or 231-NR4A1#2 cells were injected into the second pair of mammary fat pads of 6-week-old female SCID mice. Each site was injected with 2 million cells in 100 μl of serum-free DMEM medium. Injected mice were maintained for tumor growth. The tumor length (L) and width (W) were measured once a week and tumor volume was estimated by the formula of (L×W^2^)/2. Mice were euthanized at different time points or when their tumor sizes reached about 2 cm^3^. Tumor tissue and lung tissue were collected, fixed, dehydrated and embedded as described in the preceding section of “Mouse models and mouse tissue sampling”. Tumor sections were prepared for Ki67 IHC using a Ki67 antibody (#550609, BD, Biosciences, Biosciences, San Jose, CA). Lung sections were prepared for H&E staining.
